# The Peritoneal Membrane—A Potential Mediator of Fibrosis and Inflammation among Heart Failure Patients on Peritoneal Dialysis

**DOI:** 10.3390/membranes12030318

**Published:** 2022-03-11

**Authors:** Margarita Kunin, Pazit Beckerman

**Affiliations:** Sheba Medical Center, Institute of Nephrology and Hypertension, Sackler Faculty of Medicine, Tel-Hashomer, Tel Aviv-Yafo 52621, Israel; pazit.beckerman@sheba.health.gov.il

**Keywords:** peritoneal dialysis, congestive heart failure, peritoneal membrane

## Abstract

Peritoneal dialysis is a feasible, cost-effective, home-based treatment of renal replacement therapy, based on the dialytic properties of the peritoneal membrane. As compared with hemodialysis, peritoneal dialysis is cheaper, survival rate is similar, residual kidney function is better preserved, fluid and solutes are removed more gradually and continuously leading to minimal impact on hemodynamics, and risks related to a vascular access are avoided. Those features of peritoneal dialysis are useful to treat refractory congestive heart failure patients with fluid overload. It was shown that in such patients, peritoneal dialysis improves functional status and quality of life, reduces hospitalization rate, and may decrease mortality rate. High levels of serum proinflammatory cytokines and fibrosis markers, among other factors, play an important part in congestive heart failure pathogenesis and progression. We demonstrated that those levels decreased following peritoneal dialysis treatment in refractory congestive heart failure patients. The exact mechanism of beneficial effect of peritoneal dialysis in refractory congestive heart failure is currently unknown. Maintenance of fluid balance, leading to resetting of neurohumoral activation towards a more physiological condition, reduced remodeling due to the decrease in mechanical pressure on the heart, decreased inflammatory cytokine levels and oxidative stress, and a potential impact on uremic toxins could play a role in this regard. In this paper, we describe the unique characteristics of the peritoneal membrane, principals of peritoneal dialysis and its role in heart failure patients.

## 1. Introduction

Peritoneal dialysis (PD) is a home-based dialysis therapy for patients with end stage kidney disease (ESKD). This type of dialysis relies on the structure, physiology, and characteristics of a specialized membrane—the peritoneum. The prevalence of peritoneal dialysis varies from country to country. It accounts for approximately 11% of patients undergoing dialysis overall [1,2,3]. The advantages of peritoneal dialysis compared with hemodialysis (HD) include ease of use, less need for expert medical staff and technical support, and accessibility in remote geographical locations. PD gives patients more flexibility, allowing them to continue working; it preserves residual renal function (RRF) and has a lower cardiovascular impact [4,5,6]. There are only a few contraindications to peritoneal dialysis. These include an inadequate cognitive or physical ability of the patient or an assisting partner to learn and perform peritoneal dialysis and lack of a suitable peritoneal cavity due to extensive scarring or adhesions. In this regard, the degree of scarring could be assessed during the peritoneal cavity laparoscopic visualization at the time of attempted catheter placement and could be treated by adhesiolysis [7]. Other relative contraindications to peritoneal dialysis include active bowel diseases, for example, inflammatory bowel disease, ventriculoperitoneal shunt, severe chronic lung disease, or abdominal skin infections. Numerous studies have shown that HD and PD are associated with similar survival among patients with ESKD [8,9,10,11]. Health-related quality of life is equivalent for patients who are receiving PD and those receiving HD [12,13].

## 2. Dialytic Procedure

Peritoneal dialysis is performed by instilling fluid, called dialysate, into the peritoneal cavity. The fluid is allowed to dwell (the dwell is the time during which the dialysate remains in the abdominal cavity) for a defined period, after which it is drained and fresh fluid is instilled. The volume of fluid instilled is 2 L in most adults, although lower volumes are often used in smaller patients and higher volumes in larger patients. During the dwell period, solute diffusion and ultrafiltration occur across the peritoneal membrane; the used dialysate is then discarded, and the cycle is repeated. Peritoneal dialysis may be performed manually, usually four times daily, with the dialysate dwelling in the abdominal cavity between exchanges to equilibrate; this is called continuous ambulatory peritoneal dialysis (CAPD). Alternatively, a mechanical device, a “cycler”, may be used to perform a number of exchanges over a period of several hours in a procedure called automated peritoneal dialysis (APD).

## 3. Peritoneum

The peritoneum is a serosal membrane that lines the peritoneal cavity. The peritoneum approximates body surface area in size, and typically ranges from 1 to 2 m^2^ in an adult. Anatomically, it is composed of two layers: the visceral peritoneum, which covers the abdominal organs and accounts for 80% of the total surface area, and the parietal peritoneum, which lines the undersurface of the diaphragm and the interior surface of the anterior abdominal wall [14]. During peritoneal dialysis, the major player in peritoneal transport is the parietal peritoneum, this is because only approximately one-third of the visceral peritoneum is in contact with the dialysis solution during PD treatment [15]. The peritoneal cavity contains the omentum, ligaments, mesentery, and intraperitoneal organs including the stomach, spleen, liver, parts of small bowel, and sigmoid colon. Retroperitoneal organs include the aorta, esophagus, the parts of the small bowel and the colon, pancreas, kidneys, ureters, and adrenal glands. The visceral peritoneum receives its blood supply from the superior mesenteric artery, and its venous drainage through the portal system. The parietal peritoneum receives blood from the lumbar, intercostal, and epigastric arteries and drains into the inferior vena cava. The total peritoneal blood flow ranges from 50 to 150 mL/min². The main lymphatic drainage of the peritoneum and of the peritoneal cavity is the subdiaphragmatic lymphatic system, which drains into the right lymphatic duct through large collecting ducts. Additional lymphatic drainage occurs via smaller lymphatics in the visceral and the parietal peritoneum. Histologically, the peritoneum consists of a single layer of mesothelial cells resting on submesothelial interstitial tissue, a gel-like matrix containing fibroblasts, adipocytes, collagen fibers, nerves, lymphatic vessels, and capillaries [16].

## 4. Peritoneal Transport

**Figure 1:**
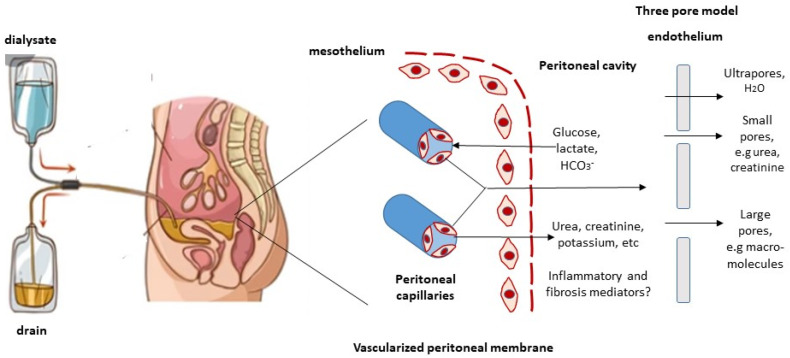
Peritoneal membrane transport in PD. During peritoneal dialysis exchanges, dialysate is instilled into the peritoneal cavity, and is later drained. During dwells, transport of water, solutes, and molecules occurs across the peritoneal membrane through diffusion and convection. According to the three-pore model, transport depends on the relative abundance of large and small pores (endothelial clefts) and ultrapores (aquaporins). It is not known whether macromolecules such as cytokines or fibrosis mediators are cleared by the peritoneal membrane.

During peritoneal dialysis, transport of solutes and water occurs across the peritoneal membrane, between two fluid-containing compartments: on one side, the peritoneal capillaries, which contain high levels of waste products such as urea, creatinine and potassium, and on the other side, the dialysis solution in the peritoneal cavity. The dialysis solution typically contains sodium, chloride, and lactate or bicarbonate, and is rendered hyperosmolar by a high concentration of glucose (Figure 1). Three transport processes occur simultaneously during a peritoneal dialysis dwell: diffusion, ultrafiltration, and absorption. In PD, fluid (dialysate) is instilled in the peritoneal cavity, and solutes diffuse from the blood in the peritoneal capillaries into the dialysate [17]. Similarly, imposition of a transmembrane pressure gradient creates the driving force for ultrafiltration of fluid from the capillaries into the dialysate. Peritoneal dialysis involves osmotic pressure created by the intraperitoneal instillation of hypertonic dialysate, usually as glucose in the form of 1.5, 2.5, or 4.25% dextrose. Higher concentrations of glucose exert higher osmotic pressures and effect greater degrees of ultrafiltration [17]. Over time, glucose diffuses from dialysate into the peritoneal capillaries, which leads to dissipation of the osmotic gradient and slows the rate of ultrafiltration. Solute transfer across the peritoneal capillaries is bidirectional. Solutes such as urea, creatinine, and potassium diffuse from the bloodstream into the dialysate, whereas glucose diffuses from the dialysate into the peritoneal capillaries.

### 4.1. The Three-Pore Model

The three-pore model has been well validated by clinical observations. According to this model, the movement of solutes and water across the peritoneal capillary depends on the relative abundance of three kinds of pores, based on their different sizes [18,19,20] (Figure 1).

Large pores have a radius of 20–40 nm, they are formed by clefs between the endothelial cells and are small in number. Macromolecules can be transported by convection through these pores.

Small pores have a radius of 4–6 nm, they are also formed by clefts between endothelial cells. This type of pore is the most abundant type, accounting for more than 90% of the transport of small solutes (e.g., urea, creatinine, sodium, and potassium), which is associated with water removal.

Ultrapores have a radius of <0.8 nm and are comprised of aquaporin channels in the endothelial cell membrane. Only water is transported through these ultrapores. The transport of water via aquaporin-1 contributes to around 50% of ultrafiltration in PD.

The rate of solute transfer across the peritoneum depends on the concentration gradient and the degree of peritoneal vascularity [21], which varies from person to person. Thus, peritoneal transport is dependent on the surface area of the peritoneal capillaries rather than on the total peritoneal surface area. The distance of each capillary from the mesothelium determines its relative contribution. The term “effective peritoneal surface area” refers to the area of the peritoneal surface that is sufficiently close to the peritoneal capillaries to play a role in peritoneal transport. In patients with less peritoneal vascularity, solutes diffuse slowly in both directions. Waste products transfer slowly into the dialysate, and the glucose gradient that is driving ultrafiltration dissipates slowly. Conversely, in patients with greater peritoneal vascularity, solutes diffuse more rapidly, also in both directions. Waste products accumulate in the dialysate more rapidly, and the glucose gradient dissipates more rapidly. Such patients have poor, sometimes even negative ultrafiltration, especially during long dwells. The use of a non-glucose-based fluid such as icodextrin during long dwells may be beneficial in these patients [22]. Icodextrin is a colloid osmotic agent that does not diffuse across the peritoneum; its effect on ultrafiltration is sustained for 12 to 16 h [23]. Icodextrin has been shown to improve ultrafiltration and volume status in PD patients [24]. It has also been shown to improve glycemic control, decrease weight gain, and lessen glucose-induced lipid abnormalities [25,26]. There is some evidence of better long-term preservation of peritoneal membrane function [27]. Other types of dialysate fluids include an amino acid-based fluid and fluids that are low in glucose degradation products (GDPs). So-called “biocompatible” solutions are two-bag system solutions that have a physiological pH after mixing. They contain reduced amounts of GDPs and they are theoretically more biocompatible than standard solutions where the pH is 5.5. However, there is a lack of consistent evidence supporting superiority of those solutions compared to conventional PD solutions, in preservation of membrane function and in long-term survival of patients or technique.

### 4.2. Removal of Middle Molecules and Protein-Bound Solutes

Several factors affect the rate of solute transport through the peritoneal membrane, of which the molecular weight (MW) of the solutes is likely the most important factor affecting transport rate [18,19,20,28]. Peritoneal transport of larger molecules occurs at a much slower rate compared to small solutes. Thus, creatinine (MW 113 g/mol) is slower than urea (MW 56 g/mol), inulin (MW 5200 g/mol) is slower than creatinine, and larger proteins cross the peritoneum very slowly. 

Proteins with higher molecular weights, such as albumin, transferrin, and immunoglobulin G (IgG), utilize the large pores described above for transport across the peritoneal membrane and into the peritoneal cavity. The exact mode of this process is a subject of debate, but both size-selective diffusion and convection seem to contribute [29]. Regardless of the mechanism, this process is sufficiently slow that serum proteins are present in low concentration in the dialysate, and equilibration with the plasma does not occur at clinically used dwell times. As a result, the clearance of these molecules approximates their mass transfer area coefficient (MTAC). The transport of these solutes out of the peritoneal cavity occurs mainly via the subdiaphragmatic lymphatics and, to a lesser degree, the peritoneal interstitium [30,31]. This process is independent of molecular size [31].

Even through serum proteins with higher molecular weights, such as albumin, transport slowly, the daily peritoneal protein loss with PD could be substantial, averaging 6–8 g, and is significantly increased during episodes of peritonitis. As a result of this daily loss, serum albumin decreases in patients starting PD. It is currently unclear whether the lower serum albumin in patients treated with PD puts them at any risk compared to patients undergoing hemodialysis. 

The dialysate to plasma concentration ratio (D/P) of middle-sized and large solutes correlated positively with D/P of creatinine, a small molecule that easily transports across the peritoneum. Peritoneal clearances of large uremic toxins, such as beta2-microglobulin and p-cresol, are significantly lower as compared to the clearances of urea nitrogen and creatinine [32]. Beta2-microglobulin has a high molecular weight (11.8 kDa), which interferes with its diffusive and convective transport across the pores of the peritoneal membrane. Therefore, as opposed to small water-soluble molecules, the peritoneal clearance of beta2-microglobulin depends largely on the total dwell hours of peritoneal dialysis treatment, rather than on the number of exchanges per day [33].

However, molecular weight cannot fully explain the features of peritoneal permeability of all molecules [34]. Other factors, including charge and protein binding rate, may impact peritoneal transport rates. Since the tissue mass of the peritoneal cavity is smaller relative to the entire body, concentrations of serum cytokines, growth factors, cardiac markers, and adipokines are usually higher than those in the dialysis fluid by factors of 10–100 [35]. There was no inverse correlation between the molecular weights of these mediators and the dialysate-to-serum ratios, suggesting that their higher effluent concentrations result from significant local intraperitoneal synthesis and less from size-based peritoneal transport [36]. 

## 5. Molecular and Genetic Studies

Among patients starting treatment with peritoneal dialysis, there is a broad variability in water and solute transport across the peritoneal membrane, which influences dialysis prescriptions and outcomes [37,38,39,40]. A recent genome-wide study showed that the peritoneal small-solute transport rate was associated with a polygenic risk score and with 17% heritability; these findings support a genetic influence on solute transport across the peritoneal membrane [41]. It was recently demonstrated that a common promoter variant rs2075574 in AQP1 was associated with decreased ultrafiltration and with an increased risk of death or technique failure among peritoneal dialysis patients [42]. The translation of genetic and molecular insights to precision medicine would help to understand better the dialysis process and to improve dialysis care [41,43].

Recent study analyzed peritoneal biopsies of patients (uremic, PD-treated, and with encapsulating peritoneal sclerosis (EPS)), and found expression of glucose transporter—sodium-glucose transporte-2 (SGLT-2), glucose transporter 1 (GLUT1), and glucose transporter 3 (GLUT3)—in the peritoneal membrane. Protein expression of SGLT-2 increased with PD duration and was significantly increased among EPS patients [40]. Preclinical studies in mice showed that SGLT-2 inhibitors or downregulation of SGLT-2 reversed pathological changes in the peritoneum. The authors therefore conclude that SGLT-2 inhibitors may have potential therapeutic benefit among PD patients. These drugs may reduce glucose absorption through the peritoneal membrane and delay the long-term deleterious effects of glucose on the transport function of the peritoneal membrane, thus prolonging its use and perhaps preventing EPS [44].

Peritoneal fibrosis is a common complication of long-term PD that ultimately leads to ultrafiltration failure and discontinuation of PD. There is currently no effective therapy to prevent or delay this pathologic process. Recent studies have reported that epigenetic modifications play an important role in PD-associated peritoneal fibrosis. Accumulating evidence suggests that epigenetic therapies may have the potential to prevent and treat peritoneal fibrosis clinically. The major epigenetic modifications reported in peritoneal fibrosis include DNA methylation, histone modification, and noncoding RNAs. The mechanisms of epigenetic regulation in peritoneal fibrosis predominantly involve modification of signaling molecules, transcriptional factors, and genes [45].

## 6. Peritoneal Membrane Preservation

Preservation of the integrity and transport function of the peritoneal membrane is fundamental to the long-term success of PD treatment. Repeated exposure of the peritoneal membrane to high and non-physiological concentrations of glucose through the peritoneal dialysis solutions results in sclerosis of the peritoneum, leading to progressive and irreversible loss of its ultrafiltration capacity. This may eventually lead to PD technique failure and switch to HD [46]. Moreover, high levels of glucose results in increased absorption into the systemic circulation. This in turn leads to hyperglycemia, obesity, and hyperlipidemia.

It is possible to assess peritoneal membrane transport function in patients on peritoneal dialysis with peritoneal equilibration test (PET) [47]. The solute transport rates are assessed by the rates of their equilibration between the peritoneal capillary blood and dialysate. The ratio of solute concentrations in dialysate and plasma (D/P ratio) at specific times during the dwell defines the extent of solute equilibration. Changes in the D/P ratio could be used to monitor peritoneal membrane function over time. It was demonstrated that D/P creatinine ratio is a strong predictor of outcomes (mortality and hospitalization) in peritoneal dialysis patients [48].

### 6.1. Glucose-Sparing Strategies

General strategies to lessen the need for hypertonic glucose use include interventions to preserve residual renal function as well as non-glucose dialysis solutions containing icodextrin or amino acids that substitute glucose-containing solutions [26,49]. Innovative strategies of UF have emphasized the potential benefits of combination of a crystalloid (glucose) and a colloid (icodextrin) osmotic agent in a variety of formulations that is capable of surpassing each of the components used separately during exchanges ranging from as short as 2 h to the extended 15 h dwell [50]. Two combinations of glucose and icodextrin have thus far been evaluated (1.36/7.5% and 2.6/6.8%) for use in the long exchange [50]. Both solutions demonstrated efficiency in sodium and water removal and have the potential for glucose sparing when used as part of the PD prescription [50]. A new peritoneal dialysis solution containing L-carnitine and xylitol was recently reported (reviewed in [51]). These two molecules have molecular weights similar to that of glucose, high water solubility, chemical stability in aqueous solutions, and osmotic properties, which render them suitable for use in PD fluids [51]. Studies on the biocompatibility of a PD solution containing carnitine or xylitol have shown a better profile than glucose-based solutions. Following favorable in vitro effects of carnitine–xylitol-containing solution, clinical study is currently underway. Use of the novel solutions proved well tolerated in all treated patients. The results indicate the non-inferiority of carnitine–xylitol-containing PD solutions compared to standard solutions in terms of outcomes, such as PD adequacy and characteristics of peritoneal transport [52].

### 6.2. Encapsulating Peritoneal Sclerosis

Encapsulating peritoneal sclerosis (EPS) is a rare but severe complication of long-term PD. It is characterized by intraperitoneal inflammation and fibrosis, which results in bowel encapsulation. EPS causes ultrafiltration failure and bowel obstruction and is associated with high morbidity and mortality. The prevalence of EPS in PD patients has been reported to range from 0.7 to 7.3% [53].

The significant risk factor for EPS is the duration of PD. The main factors implicated in its pathogenesis include exposure to hypertonic glucose-containing solutions, long duration of PD therapy, and repeated episodes of peritonitis. The two-hit hypothesis could explain the development of EPS. Peritoneal damage due to chronic exposure to unphysiological PD fluids, which results in morphologic and functional changes of the peritoneal membrane, represents the first hit [54,55]. Chronic uremia associated pro-inflammatory and oxidative stress may further accelerate these changes. Peritoneal damage at this stage is represented by mesothelial cells detachment, resulting in progressive peritoneal denudation, or by a mesothelial to mesenchymal transition (MMT). Mesothelial cells lose their polarized cytoskeletal organization and cell-to-cell contacts and acquire a myofibroblast-like phenotype [56]. Extracellular matrix compounds, profibrotic and angiogenetic cytokines are secreted by those transformed mesothelial cells [57,58]. A disproportionate decrease in ultrafiltration capacity compared with decline in solute transport (i.e., uncoupling) may identify increased EPS risk [59]. In addition, functional changes at this phase include early changes in water transport capacity [60]. The utility of biomarkers in identifying patients at risk for EPS is unclear [59,60]. Inflammatory cytokines including interleukin (IL) 6, tumor necrosis factor (TNF)-alpha, monocyte chemoattractant protein 1 (MCP-1), and others are mildly increased in patients who go on to develop clinical signs of EPS. However, there is marked variability in concentrations, and their predictive ability has not been demonstrated. An increase in fibrinogenesis and in endothelial permeability causes fibrin deposition on the peritoneum. A major inflammatory stimulus, such as peritonitis, superimposed on the chronically injured peritoneum, serves as the second hit and induces the transformation to EPS. Another possible inflammatory stimulus includes the PD catheter itself that can trigger inflammation as a foreign material [61]. The risk of EPS is found to be higher among transplant recipients who used to be on peritoneal dialysis compared with patients who are still on peritoneal dialysis [62,63]. The increased risk may be related to the cessation of dialysis itself. This is because fluid exchanges that are performed during peritoneal dialysis tend to wash away some of the excessive fibrin. When peritoneal dialysis is stopped, the inflammatory reaction and fibrin production continue. In addition, calcineurin inhibitors, a commonly used antirejection medication, have a profibrotic effect through the upregulation of TGF-β1 and other profibrogenic factors. It was also demonstrated that certain polymorphisms in genes involved in inflammation, angiogenesis, and fibrosis may increase the susceptibility of a PD patient toward developing EPS [64,65,66]. However, transplantation in PD patients with EPS may increase survival, and EPS is not a contraindication to transplantation. 

Four stages of EPS have been classified [67]: stage 1 (pre-EPS), stage 2 (inflammatory), stage 3 (encapsulating), and stage 4 (chronic) [68]. An abdominal CT scan is the main imaging modality used to diagnose EPS [69]. CT images could show thickened or calcified peritoneum and cocooning of the bowel enveloping tethered small-bowel loops. There are no guidelines or standard treatments for EPS. The early treatment of EPS is justified and based on glucocorticoids, tamoxifen, and nutritional support. At the late phase, surgical treatment could be tried. Regular monitoring of signs of imminent EPS is important. These include: high transporter status on PET, loss of peritoneal water transport, and declining UF, as well as unexplained abdominal pain, together with a reduced food intake. In such patients, switching to HD should be considered. However, the decision to stop peritoneal dialysis must be individualized and is only made after careful consideration of the risks and benefits of peritoneal dialysis versus hemodialysis. Some works demonstrated that minimization of dialysate glucose administration and use of “biocompatible” PD fluids is associated with less peritoneal membrane fibrosis and vascular sclerosis through suppression of advanced glycation end-product accumulation [70]. While the multicenter prospective observation study (the NEXT-PD study) confirmed fewer cases of EPS [71], other studies did not.

## 7. Peritoneal Dialysis in Congestive Heart Failure

Severe congestive heart failure (CHF) is often accompanied by kidney dysfunction. The term cardiorenal syndrome has been applied to the presence or development of functional renal dysfunction in patients with heart failure. Patients with severe CHF commonly develop diuretic resistance. Chronic heart failure patients with severe symptoms despite maximum guideline-directed medical therapy are classified by the American College of Cardiology Foundation/American Heart Association as having stage D heart failure [72]. Peritoneal ultrafiltration (UF) allows at-home daily fluid removal. Peritoneal UF has many advantages compared to hemodialysis-based UF: it has a very low impact on hemodynamics, residual kidney function is better preserved, normonatremia is maintained through sodium sieving, better removal of ascites fluid, elimination of vascular access complications, and perhaps improved systemic inflammation [73,74]. It was demonstrated that PD was not associated with myocardial stunning, described in HD [75]. We and others have demonstrated that in refractory CHF patients PD improved the functional status and quality of life, reduced hospitalization rate, and may even decrease mortality rate [76,77,78,79]. We also showed that CHF patients treated with PD had similar survival compared to CHF patients treated with HD, however, the clinical profiles of those two groups were different [80]. CHF patients treated with PD were younger, had lower blood pressure, more severe heart disease per echocardiography, and higher eGFR compared to those treated with HD. This difference may be explained by the fact that more debilitated, hemodynamically instable CHF patients are preferentially referred to PD. Another advantage of PD compared to HD in refractory CHF is the possibility to adjust the treatment program to patient needs. Some patients require only a few PD exchanges per day for fluids removal, while others, with advanced kidney failure, should be treated with a full dialysis program for both fluid and solutes removal. Since many refractory CHF patients starting PD for fluid removal have substantial residual renal function incremental dialysis may be used. Incremental PD is defined as less than “standard dose” PD prescription [81]. There are many potential advantages of incremental PD, including reduced peritonitis risk, lower peritoneal glucose exposure and cost, preservation of residual renal function, and improved quality of life [81]. PD dose is increased as needed if and when residual renal function declines. The main clinical picture of some CHF patients is refractory ascites. We demonstrated that those patients could be treated with at-home drainage of ascites via Tenckhoff catheter [82]. Following regular massive ascites removal, those patients showed significant improvement in functional status, improved kidney function, less systemic volume overload, and improved inflammatory state compared to their baseline status. Large-volume ascetic fluid removal can lead to reduction of intra-abdominal pressure and improvement in venous congestion and renal function [83]. Increased venous return following ascites drainage augments cardiac filling and by this improves cardiac function [84]. Regular frequent ascites removal by a peritoneal catheter may be better than intermittent paracentesis on as-needed basis, as it provides a more favorable and prolonged effect on the intrathoracic pressure [83].

CHF is a complex multifactorial disease. Among other factors, inflammation and fibrosis play an important part in disease pathogenesis and progression. Other than diffusive and convective properties, it is intriguing to speculate that the peritoneum takes part in the clearance of inflammatory and pro-fibrotic mediators, thus directly affecting disease course. This hypothesis was tested in the following studies.

### 7.1. Inflammation

**Figure 2:**
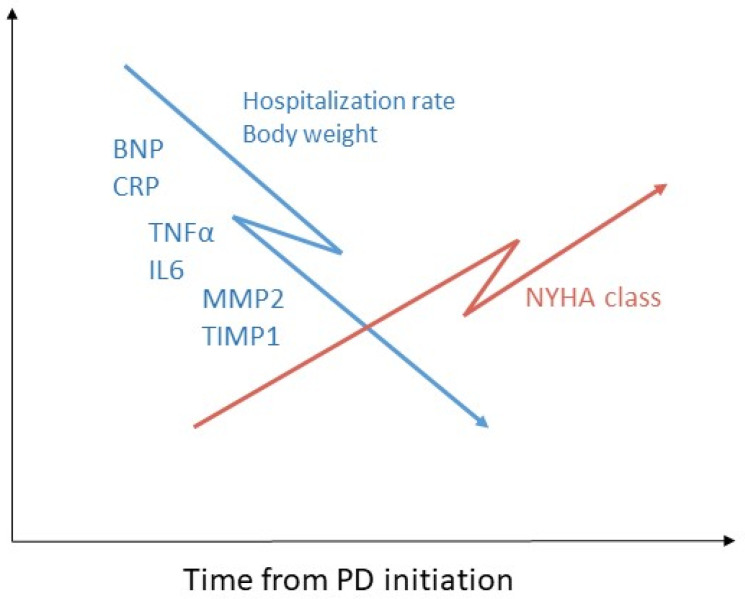
Changes in clinical outcomes, inflammatory mediators, and fibrosis markers in congestive heart failure (CHF) patients during peritoneal dialysis treatment. Patients with CHF who are treated with peritoneal dialysis for refractory volume overload experience clinical improvement (increased fluid removal, decreased body weight, decreased rate of re-admissions and improvement in functional NYHA class), and a decrease in brain natriuretic peptide (BNP), as well as decrease in inflammatory (CRP, TNFα, and IL6) and fibrosis (MMP2, TIMP1) mediators. Whether these mediators decreased because of improved systemic hemodynamics or due to clearance across the peritoneal membrane is still unknown.

Recent evidence suggests that pro-inflammatory cytokines play a pathogenic role in CHF. Inflammatory cytokines may alter myocardial functions by several mechanisms, both direct and indirect. Direct mechanisms include induction of hypertrophy and fibrosis, decrease in cardiac contractility via changes in intracellular calcium transport and signal transduction via β-adrenergic receptors, apoptosis, and upregulation of myocardial remodeling genes [85]. Indirect effects include bone marrow dysfunction resulting in secondary anemia and aberrant activation of endothelial cells and peripheral muscle resulting in induction of inflammation [85]. It was previously demonstrated that circulating levels of proinflammatory cytokines TNF-α and IL6 are increased in CHF and correlate with disease severity [85]. We investigated the effect of peritoneal dialysis treatment on levels of inflammatory cytokines among refractory CHF patients [74] (Figure 2). All patients were already receiving maximally tolerable drug therapy according to the heart failure guidelines. Following PD initiation, the patients demonstrated significant clinical improvement, manifested by improved volume control and NYHA functional class. Brain natriuretic peptide (BNP) levels significantly decreased and remained stable after 3 months and 6 months of PD treatment, respectively. C-reactive protein, a known plasma inflammatory marker, as well as circulating proinflammatory cytokines TNF-α and IL-6, also decreased significantly 3 and 6 months after PD treatment initiation [74]. Thus, PD provided additional benefit to maximal conventional drug treatment in severe CHF patients. The favorable effect of PD on inflammatory state in refractory CHF patients is even more interesting considering the fact that peritoneal dialysis may by itself contribute to systemic inflammation [86]. Prolonged exposure to dialysis fluid with a high glucose concentration and glucose degradation products (GDPs), loss of residual renal function, and increased body fat mass all could intensify systemic inflammation in PD patients [86]. Whether clearance of proinflammatory cytokines by the peritoneal membrane significantly affects cytokine plasma levels is still unknown. Clearances of high molecular weight compounds (e.g., b2-microglobulin) by PD are known to be significantly lower compared to smaller compounds such as urea, nitrogen, and creatinine. This is because high molecular weight disrupts diffusive and convective transport across peritoneal membrane pores [32]. Previous work suggested that ultrafiltration alone cannot remove high molecular weight substances including cytokines in clinically relevant amounts due to the intrinsic parameters of UF [87]. TNF-α and IL-6 have a high molecular weight (approximately 26 kDa and 24 kDa). Since most of our patients require only 1–2 short exchanges per day, it could be assumed that significant removal of cytokines by this PD program is unlikely. Whether more intensive PD programs result in higher cytokine clearance and lower serum levels is yet to be determined. It was suggested that there are interactions between the renin-angiotensin system, the adrenergic system, and the activity of proinflammatory cytokines. Therefore, some of the conventional therapies for CHF may be beneficial, at least partly through the modulation of proinflammatory cytokines. We assume that fluid removal during PD results in a decrease in neurohumoral activation, leading to a more physiological set-point, further leading to a reduction in proinflammatory cytokines. 

### 7.2. Fibrosis

Fibrosis is a common pathogenetic mechanism in cardiomyopathies such as dilated and hypertrophic cardiomyopathy, and in myocardial infarction [88]. Clinically, cardiac fibrosis manifests with diastolic dysfunction, arrhythmias, and sudden death [89]. Biochemically, cardiac tissue repair and fibrosis can be monitored using known biomarkers of collagen turnover. It was assumed that serum biomarker excreted into the circulation reliably reflects the accumulation of fibrous tissue in myocardium. Such circulating markers have been previously used in experimental models as well as in patients [89]. We examined the effect of PD treatment on the levels of circulating fibrosis markers among refractory CHF patients [90] (Figure 2). Markers tested included procollagen type III C-peptide (PIIINP), matrix metalloproteinase 2 (MMP-2), and tissue inhibitor of metalloproteinases I (TIMP-1). Levels were measured at baseline and after 3 and 6 months of PD treatment in CHF patients who started PD in the indication of fluid overload. Following PD initiation, patients demonstrated significant clinical improvement, manifested by improved volume control and NYHA functional class and decreased hospitalization rate and serum BNP levels. Serum MMP-2 and TIMP-1 decreased significantly during PD treatment, while circulating PIIINP did not show a consistent pattern, and either decreased or increased. Patients whose circulating PIIINP levels decreased had a more severe CHF: baseline serum albumin and baseline mean arterial blood pressure were significantly lower, serum CRP was higher, and the decrease in their hospitalization rate was less pronounced compared to the patients whose circulating PIIINP increased. The possible explanation of this effect of PD on circulating PIIINP levels could be higher metabolic rate and more pronounced myocardial fibrosis deposition in CHF patients with more advanced disease. In cases where all three markers decreased, there was a trend towards longer survival compared to patients in whom markers increased or did not change [90]. Since serum MMP-2 and TIMP-1 decreased significantly and serum PIIINP did not change significantly in the entire cohort, it could be assumed that PD treatment in CHF patients has a more profound effect on collagen degradation than on collagen synthesis rate. In our study, TIMP-1 demonstrated the most significant decrease following PD treatment. It was shown that angiotensin II stimulates collagen synthesis and regulates collagen degradation by enhancing TIMP-1 production in endothelial cells, thereby attenuating interstitial MMP-1 activity [91,92]. Fluid removal during PD treatment could improve neurohumoral activation, leading towards a more physiological state and this effect may be responsible for the observed decrease in TIMP-1 in our work. 

As with the proinflammatory cytokines, the question is whether clearance through PD contributes to the decrease in circulating fibrosis markers levels. PIIINP (42 kDa), TIMP-1 (28 kDa), and MMP-2 (72 kDa) have a high molecular weight. It is therefore unlikely that the partial PD program prescribed to our patients for their volume overload (1-2 exchanges a day) had a significant effect on fibrosis markers levels. Other possible explanations for the beneficial effect of PD on cardiac fibrosis include improvement in neurohumoral activation after fluid removal leading towards a more physiological state, decreased remodeling secondary to lower mechanical pressure on the heart, decreased inflammation, and possibly the clearance of uremic toxins [93,94]. We suggest that in some refractory CHF patients who suffer from significant fluid overload, PD treatment can result in a decrease in circulating fibrosis markers levels, thus augmenting the therapeutic effect of common drug regimens. 

## 8. Conclusions and Future Research

The peritoneum is a specialized endogenous membrane with unique structural and physiological features. It enables effective and convenient home-centered dialysis treatment, which has been shown to be especially beneficial among CHF patients.

Not all functions of the peritoneal membrane are known. Factors contributing to transport rate, other than MW of the solute should be investigated, in different dialysis patient populations. Perhaps longer or more frequent dwells could more efficiently clear high molecular weight molecules, such as inflammatory mediators, and directly affect the progression of diseases such as CHF. Molecular and genetic studies, aiming to explain variability in water and solute transport across the peritoneal membrane, could help in developing an individualized approach to the treatment. Future research should also focus on means of preservation of peritoneal membrane. In this regard, glucose-sparing peritoneal dialysis solutions should be studied.

The mechanisms that underlie the beneficial effect of PD in refractory CHF patients are still unclear. Potential mechanisms include maintenance of fluid balance leading to neurohumoral activation resetting towards a more physiological condition, decrease in mechanical pressure on the heart leading to blunting of remodeling, reduced inflammatory cytokine profile and oxidative stress, and a potential impact on uremic toxins. Additional studies are needed in refractory CHF with volume overload to compare PD treatment with other modalities such as HD.
